# Global Benchmark Values for Laparoscopic Roux-en-Y-Gastric Bypass: a Potential New Indicator of the Surgical Learning Curve

**DOI:** 10.1007/s11695-020-05030-0

**Published:** 2020-10-13

**Authors:** Guillaume Giudicelli, Michele Diana, Mickael Chevallay, Benjamin Blaser, Chloé Darbellay, Laetitia Guarino, Minoa K. Jung, Marc Worreth, Daniel Gero, Alend Saadi

**Affiliations:** 1 Department of Surgery, Neuchâtel Hospital, Rue de la Maladière 45, 2000 Neuchâtel, Switzerland; 2 Division of Visceral Surgery, Department of Surgery, Geneva University Hospital, Rue Gabrielle-Perret-Gentil 4, 1211 Geneva 14, Switzerland; 3 IRCAD, Research Institute against Digestive Cancer, Strasbourg, France; 4 Department of Surgery, Strasbourg University Hospital, 1 Place de l’Hôpital, 67000 Strasbourg, France; 5 Department of Visceral Surgery, Lausanne University Hospital, Rue du Bugnon 46, 1011 Lausanne, Switzerland; 6 Department of Surgery and Transplantation, University Hospital Zurich, Rämistrasse 100, 8091 Zurich, Switzerland

**Keywords:** Global benchmark, Laparoscopic Roux-en-Y-gastric bypass, Learning curve

## Abstract

**Background:**

Laparoscopic Roux-en-Y gastric bypass (LRYGB) is a technically demanding procedure. The learning curve of LRYGB is challenging and potentially associated with increased morbidity. This study evaluates whether a general laparoscopic surgeon can be safely trained in performing LRYGB in a peripheral setting, by comparing perioperative outcomes to global benchmarks and to those of a senior surgeon.

**Methods:**

All consecutive patients undergoing primary LRYGB between January 2014 and December 2017 were operated on by a senior (A) or a trainee (B) bariatric surgeon and were prospectively included. The main outcome of interest was all-cause morbidity at 90 days. Perioperative outcomes were compared with global benchmarks pooled from 19 international high-volume centers and between surgeons A and B for their first and last 30 procedures.

**Results:**

The 213 included patients had a mean all-cause morbidity rate at 90 days of 8% (17/213). 95.3% (203/213) of the patients were uneventfully discharged after surgery. Perioperative outcomes of surgeon B were all within the global benchmark cutoffs. Mean operative time for the first 30 procedures was significantly shorter for surgeon A compared with surgeon B, with 108.6 min (± 21.7) and 135.1 min (± 28.1) respectively and decreased significantly for the last 30 procedures to 95 min (± 33.7) and 88.8 min (± 26.9) for surgeons A and B respectively.

**Conclusion:**

Training of a new bariatric surgeon did not increase morbidity and operative time improved for both surgeons. Perioperative outcomes within global benchmarks suggest that it may be safe to teach bariatric surgery in peripheral setting.

## Introduction

Bariatric surgery improves obesity-related comorbidities and quality of life. It is also the only effective long-term treatment of morbid obesity [1–5]. Although laparoscopic sleeve gastrectomy is the most common bariatric procedure worldwide [6], gastric bypass is the preferred procedure in Switzerland, accounting for 74% (4083/5491) of all bariatric procedures in 2016 [7]. The laparoscopic method is the gold standard given its benefits over open surgery [8–11] and is technically demanding. To reduce perioperative complications, the learning curve of newly trained bariatric surgeon requires attention [11–14]. Competence refers to an improvement in operative parameters [15] and previous studies suggest that the learning curve includes 100 cases to reach a significant reduction in operative time and morbidity [16–19]. Maintaining operative time and complication rate, despite more complicated cases [15], is considered mastery and requires approximately 500 cases [20]. However, this number may vary from surgeon to surgeon and does not account for other relevant perioperative outcomes.

A new approach has been recently introduced in surgical outcome reporting, referred to as global benchmarks [21]. Benchmarking is a well-standardized quality enhancement process from the realm of manufacturing and economy, which uses best performance in a given field as a reference point for others to improve. Gero et al pooled consecutive low-risks cases from international high-volume centers, and relevant outcome indicators were presented as median and interquartile range [22]. The best achievable results in bariatric surgery were arbitrarily set at the 75th percentile meaning that morbidity below this value is considered acceptable, whereas morbidity above this cutoff should warrant initiatives for improvement [22]. The established global benchmarks [21] allow unbiased comparison of surgical outcomes in similarly low-risk patients across centers. The objectives of this study were as follows: (1) to evaluate whether a skilled laparoscopic surgeon can be safely trained in performing LRYGB by comparing perioperative outcomes to recently introduced global benchmarks for LRYGB, and (2) to evaluate the effect of a fixed team approach on perioperative outcomes on a center level.

## Materials and Methods

### Study Population and Design

This study was approved by the institutional review board of Geneva University Hospital, Switzerland (number: 2019-00691), and informed consent does not apply. All consecutive patients undergoing a primary LRYGB at the Hospital of Neuchâtel, a regional non-university teaching hospital in peripheral setting, were prospectively included from the start of the bariatric program in January 2014 until December 2017. All patients met the International Federation of Surgery for Obesity (IFSO) [23] criteria for bariatric surgery. Patients with a body mass index (BMI) > 50 kg/m^2^ or with a previous history of bariatric or gastric surgery, or undergoing associated surgical procedures except for cholecystectomy, were excluded.

A comparison of perioperative outcomes with global benchmarks in bariatric surgery was performed for all patients operated on by each surgeon and in a subgroup of “low-risk” patients according to previously published criteria, allowing unbiased comparison of subgroups [22]. Patients excluded from the “low-risk” group had a history of previous intra-abdominal surgery, cardiovascular disease (e.g., cardiac arrhythmia, stroke, coronary artery disease), history of thromboembolic events and/or therapeutic anticoagulation, diabetes mellitus (type 1 and type 2, as defined by the American Diabetes Association), obstructive sleep apnea (recurrent episodes of upper airway collapse during sleep), chronic obstructive pulmonary disease (FEV1/ FVC < 0.7), chronic kidney disease (eGFR < 30 mL/min/1.72 m2), inflammatory bowel disease (ulcerative colitis, Crohn’s disease), immunosuppression therapy (i.e., steroids, calcineurin inhibitors), or associated surgical procedures (i.e., cholecystectomy, hiatoplasty, liver biopsy). Costs were estimated for patients with or without any surgical complications by using the algorithm developed by Staiger et al. [24].

To evaluate the effect of the procedural volume on perioperative outcomes, cases were clustered into groups of 30 to allow for a comparison between surgeon and patient groups. The number 30 was set based on previous reports suggesting that competency for LRYGB can be achieved after 100 procedures [16–18, 25]. A surgeon performing the first 30 procedures should therefore be in the early stage of his/her learning curve.

### Previous Experience in Laparoscopy and Bariatric Surgery

All procedures were performed either by the senior surgeon (A) or by the newly trained surgeon (B). Both surgeons had a wide experience in advanced laparoscopic procedures and upper GI surgery, including fundoplication, revisional hiatal surgery, and esophagectomy. Additionally, surgeon A had previously performed over 300 open then laparoscopic gastric bypass procedures at another institution before the study period.

### Learning Curve Evaluation

The main outcome of interest was all-cause postoperative morbidity up to 90 days. Other parameters to assess the learning curve were all-cause morbidity during index admission, operative time, conversion rate to open surgery, reoperation rate, length of hospital stay, and readmissions. Postoperative complications were reported in accordance with the Clavien-Dindo classification [26]. Any complication scored as Clavien-Dindo grade I or higher was reported. Percentages of total weight loss (%TWL) at 12 months and overall mortality rate were also reported.

### Surgical Technique and Perioperative Management

All patients were screened preoperatively by a multidisciplinary bariatric team and written informed consent was obtained. Esophagogastroduodenoscopy, assessment for sleep apnea and gallstones using ultrasonography were performed. Patients with gallstones underwent concomitant cholecystectomy.

All LRYGB procedures were standardized and did not change over the study period. In the operating room, the patients are placed in the Lloyd-Davies position with a reverse Trendelenburg angle of 25 degrees, the operator standing between the legs of the patient, and the assistant standing on the left side. The patients are given single intraoperative intravenous doses of Cefazolin 2 g. A pneumoperitoneum of 12 mmHg is created 15 cm below the xiphoid process. Additionally, 4 ports are routinely used: two 12 mm trocars and two 5 mm trocars are introduced on the horizontal line 12 cm below the xiphoid process. The liver is lifted with a laparoscopic retractor introduced into the left subcostal 5 mm trocar. Our technique involves a division of the stomach into a 15-25 mL gastric pouch with the use of one horizontal linear stapler firing (ECHELON FLEX™ ENDOPATH® 45 mm Stapler, Ethicon Endo-Surgery Inc., Cincinnati, OH, USA) and one or two stapler firings vertically to the angle of His. The omentum is routinely transected. The proximal jejunum is brought up antecolic 100 cm from the ligament of Treitz, and an antegastric end-to-side 3 cm gastrojejunostomy (GJ) is created with a 45 mm linear stapler. The anastomosis is closed with a STRATAFIX™ (Ethicon Endo-Surgery Inc., Cincinnati, OH, USA) running suture. The alimentary limb is measured at approximately 100 cm. A side-to-side jejunojejunostomy (JJ) is created with a 45 mm stapler and closed with a STRATAFIX™ running suture. A methylene blue test is performed to test the GJ anastomosis. The jejunum between the GJ and the JJ is divided with a 45 mm stapler. The jejunojejunostomy and Petersen’s defects are closed using a permanent V-Loc™ 3/0 running suture. All patients receive subcutaneous thromboprophylaxis with a low-molecular-weight-heparin (LMWH) the day before and 6 h after surgery, according to their body weight and until 30 days after discharge. The diet progression was divided into three phases: clear liquids on the day of surgery (1 day), pureed diet (7 days), and regular bariatric diet (final). The patients were followed at regular intervals postoperatively with the first surgical outpatient visit at 10 days.

### Statistical Analysis

Data management and statistical and graphical analysis were performed using IBM SPSS Statistics Version 22.0.02 (IBM Corporation, New York, NY, USA). Statistical analysis included the use of chi-square tests for categorical variables or Fischer’s exact test, as appropriate, as well as the Mann-Whitney *U* test for continuous variables. Normal and binomial 95% confidence intervals (CIs) are presented for univariate statistics where appropriate. The LOWESS (locally weighted scatterplot smoothing) local regression was used to describe the operative time. Results are expressed as a mean with standard deviation and medians with an interquartile range. A standard alpha of 0.05 indicated statistical significance.

## Results

### Patient Characteristics

Two hundred thirty-two patients underwent LRYGB at Neuchâtel Hospital from January 2014 until December 2017 and none was lost to follow-up. 3.4% (*N* = 8/232) of the patients had a previous history of gastric banding, which was then converted to LRYGB, 3.4% (*N* = 8/232) had another concomitant procedure (salpingectomy *N* = 1/232, pelvic exploration *N* = 1/232, reduction of gastrothorax *N* = 6/232), and 1.3% (*N* = 3/232) had a BMI > 50 kg/m^2^. Those 19 patients were excluded.

Two hundred thirteen patients were included in the study population, 83.1% (177/213) were women, mean age was 41.3 years (± 11.3), and mean BMI was 41.1 kg/m^2^ (± 3.5). 28.1% (60/213) had a high blood pressure (as defined by the American Heart Association) [27], 18.3% (39/213) had diabetes mellitus (type 1 or type 2, as defined by the American Diabetes Association) [28], 0.5% (1/213) had chronic obstructive pulmonary disease (FEV1/ FVC < 0.7) [29], 46% (98/213) had obstructive sleep apnea (recurrent episodes of upper airway collapse during sleep) [30], 16.9% (36/213) were diagnosed with mild hiatal hernia before surgery based on preoperative esophagogastroduodenoscopy and did not require a surgical procedure, and 16.9% (36/213) had gallstones and underwent concomitant cholecystectomy. Baseline demographics and clinical characteristics were similar for all patients, and for the first 30 and last 30 patients operated on by surgeons A and B. The case mix of “low-risk” patients was also similar for patients operated on by surgeons A (*N* = 54/140) and B (*N* = 28/73) *p* = 0.975.

### Comparison with Global Benchmarks

Perioperative outcomes of all patients are reported in Tables 1 and 2 and were within the global benchmarks: all-cause morbidity rate at 90 days was 8% (17/213) and 95.3% (203/213) of the patients had an uneventful hospital stay after the surgery. Mean operative time was 109.8 min (± 33.9). No procedure had to be converted to open surgery. Four patients were re-operated on: two for iatrogenic small bowel perforation, one for acute bleeding located on the vertical stapling of the gastric pouch, and one for an obstruction at the level of the jejunojejunostomy. These patients were managed laparoscopically. Mean length of hospital stay was 3.1 days (± 1), four patients were readmitted within 90 days, and there was no mortality or anastomotic leak. Mean %TWL at 12 months was 34% (± 6.8). Three “non-low risk” patients required planned intensive care unit (ICU) admission for post-operative non-invasive ventilation and therefore were not considered a morbidity. This is related to the lack of intermediate care unit at our center, and none of the three admitted patients presented life-threatening condition. Considering the 82/213 “low-risk” cases and the 73/213 cases operated on by surgeon B: all outcomes were within the global benchmarks. All mortality and morbidity outcomes are reported in Tables 2 and 3, “low-risk” cases and both surgeons’ cases were within the range of global benchmarks at 90 days, except for a wound infection rate higher than 0.5%.

**Table 1:** Perioperative course

	Benchmark	Neuchâtel Low risk *N* = 82	Neuchâtel Non-low risk *N* = 131	Neuchâtel All cases *N* = 213	Surgeon A *N* = 140	Surgeon B *N* = 73
Operation duration (min)^a^	≤ 120	98.5 (± 29.6)	115.38 (± 35.4)	109.8 (± 33.9)	107.8 (± 32.5)	114 (± 36.3)
Conversion to open surgery	0	0	0	0	0	0
Intraoperative blood transfusions	0	0	0	0	0	0
Postoperative blood transfusions	≤ 2%	0	0	0	0	0
Postoperative ICU admission	≤ 0.14%	0	0	0	0	0
ICU stay in patients admitted to ICU (days)^a^	≤ 1	–	1.5 (± 1.3)	1.5 (± 1.3)	1.5 (± 1.3)	–
Hospital stay (days) ^a^	≤ 4	2.9 (± 0.67)	3.2 (± 1.1)	3.1 (± 1)	3.1 (± 1.2)	3.1 (± 0.6)
Hospital cost in CH or USA^a^	16,203 CHF or USD	16,803 CHF or USD	17,353 CHF or USD	17,122 CHF or USD	17,304 CHF or USD	16,777 CHF or USD
Hospital cost in patients with complications in CH or USA^a^	26,485 CHF or USD	23,323 CHF or USD	22,458 CHF or USD	22,712 CHF or USD	23,120 CHF or USD	20,809 CHF or USD

^a^Values are mean (SD)

*N* number of non-missing values,, *ICU* intensive care unit, *CH* Switzerland, *USA* United Sates of America, *CHF* Swiss Francs, *USD* United States Dollars

**Table 2:** Morbidity and mortality

	Until Discharge				Until 30 days				Until 90 days			
	Benchmark	Neuchâtel Low risk *N* = 82	Neuchâtel Non-low risk *N* = 131	Neuchâtel All cases *N* = 213	Benchmark	Neuchâtel Low risk *N* = 82	Neuchâtel Non-low risk *N* = 131	Neuchâtel All cases *N* = 213	Benchmark	Neuchâtel Low risk *N* = 82	Neuchâtel Non-low risk *N* = 131	Neuchâtel All cases *N* = 213
Uneventful postoperative course	> 94%	96.4%	94.7%	95.3%	> 91%	93.9%	90.9%	92%	> 90%	93.9%	90.9%	92%
Readmission	–	–	–	–	≤ 4%	2.4%	1.5%	1.9%	≤ 5.5%	2.4%	1.5%	1.9%
Reoperation	≤ 2%	1.2%	1.5%	1.4%	≤ 2.5%	2.4%	0.8%	1.9%	≤ 4%	2.4%	0.8%	1.9%
Any complication	≤ 6%	3.6%	5.3%	4.7%	≤ 9%	6.1%	9.1%	8%	≤ 10%	6.1%	9.1%	8%
Complication CD ≥ III^a^	≤ 3.5%	1.2%	2.3%	1.9%	≤ 5%	2.4%	3%	2.8%	≤ 5.5%	2.4%	3%	2.8%
Mortality	0	0	0	0	0	0	0	0	0	0	0	0
CCI in patients with complication CD ≥ II^a^	≤ 26.2	34.8	28.6	29.9	≤ 32.5	29.8	26.9	27.9	≤ 33.73	29.8	26.9	27.9
Anastomotic leak	0	0	0	0	≤ 1.1%	0	0	0	≤ 1.3%	0	0	0
Stenosis of the anastomosis	0	0	0	0	≤ 0.3%	0	0.8%	0.5%	≤ 1.2%	0	0.8%	0.5%
Postoperative bleeding	≤ 2.2%	1.2%	1.5%	1.4%	≤ 2.2%	1.2%	1.5%	1.4%	≤ 2.2%	1.2%	1.5%	1.4%
Small bowel obstruction/internal hernia	≤ 1.4%	0	0	0	≤ 2.1%	1.2%	0	0.5%	≤ 2.1%	1.2%	0	0.5%
Wound infection	≤ 0.5%	0.5%	0	0.5%	≤ 0.5%	1.2%	1.5%	1.4%	≤ 0.5%	1.2%	1.5%	1.4%
Marginal ulcer	0	0	0	0	≤ 0.3%	0	0	0	1.5%	0	0	0

^a^Values are mean

*N* number of non-missing values, *CCI* comprehensive complication index, *CD* Clavien-Dindo classification

**Table 3:** Morbidity and mortality of each surgeon

	Until Discharge			Until 30 days			Until 90 days		
	Benchmark	Surgeon A *N* = 140	Surgeon B *N* = 73	Benchmark	Surgeon A *N* = 140	Surgeon B *N* = 73	Benchmark	Surgeon A *N* = 140	Surgeon B *N* = 73
Low-risk patients	100%	38.6%	38.3%	100%	38.6%	38.3%	100%	38.6%	38.3%
Uneventful postoperative course	> 94%	95%	95.9%	> 91%	90%	95.9%	> 90%	90%	95.9%
Readmission	–	–	–	≤ 4%	2.8%	–	≤ 5.5%	2.8%	–
Reoperation	≤ 2%	2.1%	0	≤ 2.5%	2.8%	0	≤ 4%	2.8%	0
Any complication	≤ 6%	5%	4.1%	≤ 9%	10%	4.1%	≤ 10%	10%	4.1%
Complication CD ≥ III^a^	≤ 3.5%	2.1%	1.4%	≤ 5%	3.6%	1.4%	≤ 5.5%	3.6%	1.4%
Mortality	0	0	0	0	0	0	0	0	0
CCI in patients with complication CD ≥ II^a^	≤ 26.2	33.7	26.2	≤ 32.5	29.9	26.2	≤ 33.73	29.9	26.2
Anastomotic leak	0	0	0	≤ 1.1%	0	0	≤ 1.3%	0	0
Stenosis of the anastomosis	0	0	0	≤ 0.3%	0.7%	0	≤ 1.2%	0.7%	0
Postoperative bleeding	≤ 2.2%	1.4%	1.4%	≤ 2.2%	1.4%	1.4%	≤ 2.2%	1.4%	1.4%
Small bowel obstruction/internal hernia	≤ 1.4%	0	0	≤ 2.1%	0.7%	0	≤ 2.1%	0.7%	0
Wound infection	≤ 0.5%	0.7%	0	≤ 0.5%	1.4%	0	≤ 0.5%	1.4%	0
Marginal ulcer	0	0	0	≤ 0.3%	0	0	1.5%	0	0

^a^Values are mean

*N* number of non-missing values, *CCI* comprehensive complication index, *CD* Clavien-Dindo classification

### Indicators of the Learning Curve

**Fig. 1:**
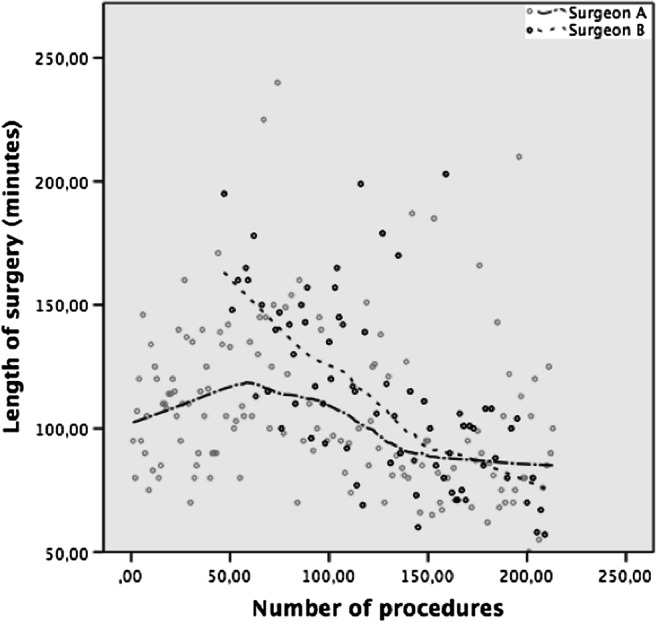
Operating time trend by LOWESS (Locally weighted scatterplot smoothing)

Table 4 summarizes indicators of the learning curve for the first and last 30 cases for each surgeon. The mean operative time for the first 30 procedures was significantly shorter for surgeon A as compared with surgeon B, with 108.6 min (± 21.7) and 135.1 min (± 28.1) respectively (*p* < 0.001). The length of surgery gradually declined with the number of operations and leveled off graphically for surgeon A, i.e., no further major changes occurring (Fig. 1). Surgeons A and B reached a similar mean operative time for the last 30 procedures of 95 min (± 33.7) and 88.8 min (± 26.9) respectively which was significantly lower compared with the first 30 procedures for both surgeons.

**Table 4:** Indicators of the learning curve

	Surgeon A *N* = 140			Surgeon B *N* = 73		
	First 30 procedures	Last 30 procedures	*p* value	First 30 procedures	Last 30 procedures	*p* value
All-cause morbidity during index admission	3.3% (1/30)	6.6% (2/30)	0.487	3.3% (1/30)	3.3% (1/30)	0.754
All-cause morbidity until 90 days	0	13.3% (4/30)	0.0384	6.7% (2/30)	3.3% (1/30)	0.553
Operative time (min)^a^	108.6 (± 21.7)	95 (± 33.7)	0.009	135.1 (± 28.1)	88.8 (± 26.9)	< 0.001
Conversion rate	0	0		0	0	.
Length of hospital stay (days)^a^	3 (± 1)	4 (± 2)	0.013	3 (± 0)	3 (± 1)	0.147
Readmission rate	0	6.6% (2/30)	0.255	0	6.6 (2/30)	0.237
Postoperative complications CD ≥ II at day 30	0	10% (3/30)	0.119	0	0	.
%TWL at 12 month^b^	31.8 (26.3–36.2)	29.6 (28.2–33.9)	0.72	34.4 (29.5–37)	39.6 (32.7–43.8)	0.012

^a^Values are mean (SD)

^b^Median (i.q.r)

*N* number of non-missing values, *%TWL* percentages of total weight loss, *CD* Clavien-Dindo classification

## Discussion

The present study compared postoperative outcomes of a senior and a newly trained bariatric surgeon in a Swiss peripheral center by using global bariatric outcome benchmarks as a reference. Both surgeons performed within the benchmark cutoffs of surgical quality and improved their operative times during the 4-year observation period. The major finding is that global outcome benchmarks can be achieved in a peripheral setting, even during the training period of a new surgeon by applying a fixed team approach.

Similar learning curve outcomes were reported for less experienced surgeons introduced to bariatric surgery: we observed an all-cause morbidity rate of 4.7% during hospital stay after the surgery, lower than the 15.8% reported by Wehrtmann et al. [15], yielding a zero mortality rate and no conversion to laparotomy. Operative time significantly declined without a loss of efficacy in weight reduction or an increased complication rate. Interestingly, the operative time of surgeon A improved even after 300 cases, supporting that 500 procedures might be needed to achieve mastery [15, 20, 25]. However, there were no significant differences comparing operative time for the last 30 procedures between surgeons A and B, suggesting that the learning curve may be shorter with a two-surgeon approach. Reames et al. reported that median operative time is independently associated with all-cause morbidity [31], and we found similar complication rates between surgeons. Anastomotic leak is potentially the most serious early complication associated with LRYGB and none was observed in this study.

Perioperative outcomes of bariatric surgery are affected by procedure-specific annual surgeons’ volume, and the number of procedures required to reach a plateau for LRYGB varies from surgeon to surgeon [32]. Defining the learning curve also depends on a wide range of parameters used to describe it [15, 18, 19]. Evidence suggests that the learning curve for LRYGB is overcome when surgical mortality is < 1%, conversion rate is 1–3%, major morbidity rate is < 5%, major leak rate is < 2%, and operating times are < 2 h [14]. However, there is no standardized marker for the evaluation of the learning curve and previous reports are often impeded by heterogeneity in case mix. In this study, we compared the postoperative course during the learning curve to global outcome benchmarks established in low-risk patients, and set at the 75th percentile of the median value of each participating academic center [21, 22]. All indicators of the LRYGB learning curve of the newly trained bariatric surgeon were within the global benchmark cutoffs, reflecting growing team experience and increased technical skills. This could indicate that the recently reported global benchmarks can be used as a reference for the learning curve of LRYGB, but may not reflect “best achievable” results. All perioperative outcomes of the low risk patients were also within the global benchmarks; overall, these findings advocate the feasibility of training a new bariatric surgeon in peripheral setting and may have implications for the credentialing of individual bariatric surgeons. In addition to the increasing caseload, a fixed team two-surgeon approach may be considered a contributor to optimal surgical outcomes. System effects seemed to reduce operative time and coaching a new surgeon positively influenced the operative time of the tutor as well.

Our study has several limitations. This was a retrospective monocentric study attempting to determine the learning curve for competence rather than mastery with a small sample size, reflecting the reality of a peripheral bariatric unit. As a result, this study includes only two surgeons which did not allow for calculation of the number of procedures needed to achieve perioperative results within global benchmarks. Also, the overall morbidity, reoperation, or readmission rates were not reduced over time. The relatively low frequency of these events indicates that to define a learning curve based on these parameters, a large number of procedures would have to be included.

Additionally, there might be other parameters potentially reflecting the learning curve which were unavailable in our data set, such as improvement of comorbidities and quality of life. This information was poorly reported and not always made available, as many patients underwent follow-up by non-surgeon healthcare providers. We chose to focus on assessing perioperative outcomes based on previously described indicators of the learning curve.

Lastly, the framework for data assessment in this study did not allow to identify a significant difference with global benchmark cutoffs value. We acknowledge that because of the small number of patients in this data set, we could only report perioperative outcomes within the benchmarks but the level of significance of this result could not be established. Likewise, a reduction in operative time related to the learning effect in the anesthesia procedure could not be established because operative time was measured from the incision to the dressing of the wound.

## Conclusion

Postoperative outcomes within global benchmarks for LRYGB may be achieved in a peripheral center during the training period of a new bariatric surgeon. LRYGB is a complex procedure and its learning curve presents a challenge in peripheral setting. From this perspective, the two-consultant approach may be an effective measure in flattening the learning curve and reducing postoperative risks.
